# New Advances in Chronic Kidney Disease: Biology, Diagnosis and Therapy

**DOI:** 10.3390/biomedicines13020518

**Published:** 2025-02-19

**Authors:** Susana Coimbra, Alice Santos-Silva

**Affiliations:** 1 UCIBIO i4HB, Faculdade de Farmácia, Universidade do Porto, Rua Jorge Viterbo Ferreira 228, 4050-313 Porto, Portugal; 2 UCIBIO i4HB, Translational Toxicology Research Laboratory, University Institute of Health Sciences (1H-TOXRUN, IUCS-CESPU), Avenida Central de Gandra 1317, 4585-116 Gandra, Portugal

Chronic kidney disease (CKD) is characterized by a progressive and usually irreversible deterioration of renal function. The prevalence of CKD is highest in older adults and is associated with increasing morbidity and mortality risk [1]. It was reported that 10% of the world population has CKD and is expected to become the fifth leading cause of death by 2040 [2]. Thus, CKD prevalence is increasing worldwide and is becoming a serious public health issue.

It is important to prevent renal disease development and/or, at least, slow disease progression. The early detection of renal injury is required in order to accomplish these goals. When CKD risk factors, such as arterial hypertension and diabetes, are present, rapid intervention is necessary. Many of the CKD patients are not aware of their disease and of its implications if not treated [3]. CKD, especially mild CKD, is *often asymptomatic* and undiagnosed, due, in part, to the low sensitivity of classic kidney biomarkers to identify early renal damage. A delay of one year in CKD diagnosis has been associated with a high risk of progression to severer CKD stages and to kidney failure that would require dialysis treatment or transplant [4]. Moreover, the worsening of CKD entails a high burden of comorbidities, especially in end-stage kidney disease (ESKD) patients under dialysis treatment, associating with poor outcomes.

A better understanding of the uremic milieu in CKD pathophysiology, of the mechanisms underlying CKD development and progression, and of its relationship with comorbidities will be very important. The Special Issue titled “New Advances in Chronic Kidney Disease: Biology, Diagnosis and Therapy” focuses on these aims; its main purpose is to gather new scientific data that help to elucidate these issues, contributing to a more effective way of managing CKD and identifying new therapeutic targets. This Special Issue published 16 contributions, 11 original research manuscripts, 4 review papers, and 1 systematic review, which are listed below.

Considering the mechanisms associated with CKD progression, in contribution 2 of this Special Issue, the authors reported that erythropoietin therapy, commonly used to treat CKD anemia, may induce complement activation, leading to the restriction of complement regulatory protein expression, which favors uncontrolled complement activation, contributing to tissue injury and CKD progression.

In another contribution 6, sphingolipid levels in plasma and in isolated lipoproteins were evaluated in controls, patients with CKD without diabetes, and patients with type 2 diabetes and macroalbuminuria. Data from this contribution suggest that sphingolipid measurement in lipoproteins is important to acquire a better understanding of the role of sphingolipids in kidney disease. Considering the importance of sphingolipids for cell biological responses and signaling pathways, the sphingolipids carried by lipoproteins may influence the development and progression of chronic inflammatory diseases, such as CKD.

The authors of contribution 9 report that the accumulation of protein-bound uremic toxins is associated with immune disturbances in ESKD patients on hemodialysis, suggesting the existence of a link between uremic dysbiosis and the deficiencies of adaptive immunity.

Besides diabetes and hypertension, ESKD patients have a higher risk of presenting other comorbidities, such as anemia and cardiovascular disease, which is the most common cause of mortality in these patients [5]. Comorbidities have a considerable impact on functional status and health-related quality of life in CKD patients. Depression is common in dialysis patients and appears to combine upsetting physical symptoms of ESKD with psychological distress, impairing significantly patients’ quality of life.

In contribution 7, the authors evaluated the impact of plasma neurofilament light chain (NfL) and brain-derived neurotrophic factor (BDNF) on the risk of depression in hemodialysis patients; although no connection between them and depression was found, a negative association between depression risk and the length of dialysis treatment was observed. Moreover, the Beck Depression Inventory score correlated negatively with the urea reduction ratio and positively with C-reactive protein, indicating the relationship of depression development with inflammation and the efficiency of dialysis.

Anemia is frequently found in CKD, contributing to impair patients’ quality of life [6]. Contribution 13 reviewed the pathophysiology, diagnosis, and treatment of anemia in CKD; this knowledge is vital for the successful diagnosis and management of anemia in CKD patients. ESKD patients commonly exhibit chronic inflammation [7]. Inflammation can be a trigger and/or consequence of CKD; it may result from the primary cause of CKD, such as in diabetes and hypertension, and may be favored by renal dysfunction changes (e.g., uremia, oxidative stress, and metabolic acidosis). In contribution 4, the authors found that the CC genotype for the *TNFRSF1B* rs3397 polymorphism was associated with decreased levels of inflammatory biomarkers and a less severe anemic condition, suggesting a more favorable inflammatory response for this genotype that probably favors erythropoiesis improvement.

The appropriate treatment and management of CKD and its comorbidities are important in order to slow disease progression, and the investigation of novel therapeutic approaches should be encouraged. In accordance, in contribution 3, treatment with extracellular vesicles derived from human liver stem cells (HLSC-EVs) was found to improve cardiac function, showing anti-fibrotic effects on the cardiac and renal tissue of a murine model of partial nephrectomy; HLSC-EVs presented the potential to counteract CKD development and to ameliorate cardiomyopathy.

Contribution 14 also provides a revision on the knowledge concerning CKD treatment, highlighting the novel therapeutic approaches that can be used in the management of this condition.

In contribution 5, the authors emphasize the importance of the early identification of arteriovenous fistula failure and of using intraoperative Doppler ultrasound-guided procedures to improve autogenous arteriovenous fistula outcomes. The ω3-poly unsaturated fatty acids (PUFAs) were found to protect tissues against injury in many disease models [8]; in contribution 8, ω3-PUFAs revealed, in an in vitro model, capacity to promote autophagic cell activation, favoring a reno-protective response against kidney injury.

Classic biomarkers of kidney function only increase when renal function is significantly reduced, allowing diagnosis only when several renal injuries have already occurred. The identification of biomarkers of cardiorenal syndrome, early diagnosis and progression of CKD and morbidity/mortality risk, will help clinicians in their therapeutic decisions and in choosing earlier and more adequate therapeutic strategies, avoiding or minimizing CKD progression. The identification of biomarkers, panel of biomarkers, cardiorenal syndrome, the early diagnosis and progression of CKD, and morbidity/mortality risk will help clinicians in their therapeutic decisions and in choosing earlier and more adequate therapeutic strategies, avoiding or minimizing CKD progression. Therefore, any attempt to find useful biomarkers is of merit. According to contribution 1, low levels of irisin appear to correlate with recent kidney-related events in patients with type 2 diabetes and with asymptomatic heart failure, with a preserved and reduced/mildly reduced ejection fraction. Although *IL6* rs1800795 polymorphism was found to be a predictor of mortality in ESKD patients under dialysis [9], in contribution 4, the *IL6* rs1800796, rs1800797, and rs1554606 polymorphisms, as well as the *TNFRSF1B* rs3397, rs1061624, and rs1061622 polymorphisms, did not associate with all-cause mortality rates of patients with ESKD.

In contribution 7, the authors reported that NfL and BDNF levels were not useful as markers of depression risk in the CKD dialysis patients. However, in contribution 10, uric acid was reported to be a predictor for ischemic heart disease in early-stage CKD patients, showing the association between high uric acid levels and increased risk. In contribution 11, it was reported that, according to the random survival forest model, creatinine, age, estimated glomerular filtration rate, and the ratio of urine protein to creatinine are useful predictors of CKD progression. A review of the importance of heart rate variability, as an important tool that can be used for prognosis in dialysis patients, was presented at contribution 12. Focusing on the improved management and prognosis of renal transplant patients, contribution 15 performed a review of the markers that can facilitate the early identification of acute renal rejection.

Acute kidney injury (AKI) and CKD are known to be closely related, with one promoting the other; therefore, the accurate and early diagnostic of AKI is crucial. In contribution 16, by performing a systematic review, the authors intended to evaluate the role of microRNAs as diagnostic markers of AKI; the authors concluded that there is a knowledge gap in using microRNAs as diagnostic markers of AKI.

By addressing important issues, such as pathophysiological mechanisms, new therapeutic approaches, the relationship with comorbidities, and eventual biomarkers of disease development and progression, we believe that this Special Issue contributes to improving the knowledge on CKD, an important worldwide public health issue.
